# Ultrafast excited-state dynamics and “three-in-one” phototheranostic properties of a phenanthroline-carbolong photosensitizer†

**DOI:** 10.1039/d5sc00013k

**Published:** 2025-03-14

**Authors:** Haixia Chang, Jiang Feng, Xin-Ao Liu, Rong Miao, Taihong Liu, Liping Ding, Yu Fang

**Affiliations:** a Institute of New Concept Sensors and Molecular Materials, Key Laboratory of Applied Surface and Colloid Chemistry of Ministry of Education, School of Chemistry and Chemical Engineering, Shaanxi Normal University China liuth121@snnu.edu.cn miaorong2015@snnu.edu.cn

## Abstract

The favorable excited-state dynamics, nonlinear optics, and extraordinary phototheranostic capabilities of conjugated metallaaromatics are attractive topics of research. A promising and multifunctional photosensitizer, double-phenanthroline-carbolong DPC, was investigated comparatively. It featured strong two-photon absorption (2PA) properties within the near-infrared (NIR) range, with a maximum 2PA cross-section of ∼7417 GM at 770 nm in MeOH. Time-dependent density functional theory and ultrafast excited-state dynamics illustrated that fast charge transfer coupled with intersystem crossing to the stable triplet state outcompeted radiative decays. The “three-in-one” phototherapeutic effect included NIR-wavelength 2PA excitation, photodynamic therapy, and photothermal therapy in DPC, as illustrated subsequently. The significant contribution from the intrinsic intramolecular charge communication along the DPC skeleton provided the possibility for moderate photothermal conversion (*η* = 36.8%) and photodynamic synergistic therapy (*Φ*_Δ_ = 8.4%). Interestingly, singlet oxygen generation from DPC was also observed when irradiated at two-photon excitation wavelengths. *In vitro* experiments demonstrated the synergistic phototoxicity of DPC in 4T1 cancer cells. This work offers insights into extraordinary carbolong metallaaromatics and highlights their potential applications in the fields of nonlinear optics and multifunctional phototheranostics.

## Abstract 2

The ultrafast excited-state dynamics, NIR two-photon absorption, and extraordinary “three-in-one” phototheranostic capabilities of a phenanthroline-carbolong photosensitizer DPC was illustrated experimentally and theoretically.

## Graphical abstract

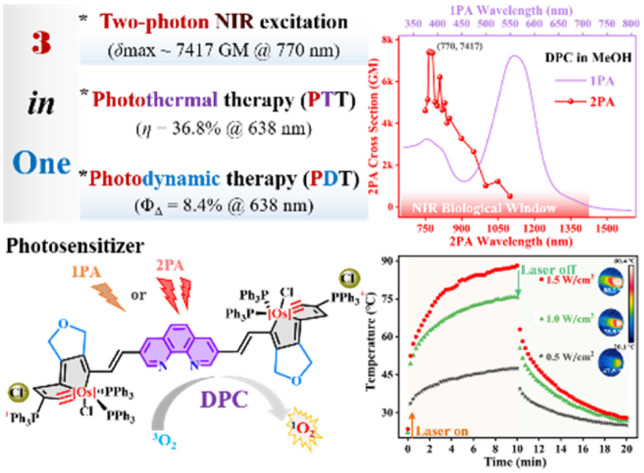

## Introduction

Phototheranostics has emerged as a promising approach to cancer therapy because of its advantages, such as minimal invasiveness, spatiotemporal accuracy, and precise controllability. The non-destructive photodynamic (PDT) and photothermal (PTT) therapies depend on photosensitizers that produce heat or reactive oxygen species when irradiated to destroy cancer cells.^1^ Compared to one-photon-absorbing photosensitizers, two-photon-exciting materials in the significant near-infrared (NIR) biological spectral window (650 ∼ 1450 nm) show distinct advantages, including deeper tissue penetration, minimal light loss, and improved phototheranostic properties.^2^ Two-photon absorption (2PA) enables the excitation of photosensitizers by longer wavelengths with lower energy and allows for higher spatial resolution due to the quadratic dependence of the 2PA rate on the incident laser light intensity.^3^ Searching for photosensitizers possessing large 2PA cross-sections (*δ*_2PA_), exceptional photostability, good biocompatibility, and high therapeutic efficacy remains a significant challenge in material science and biomedicine.

**Fig. 1:**
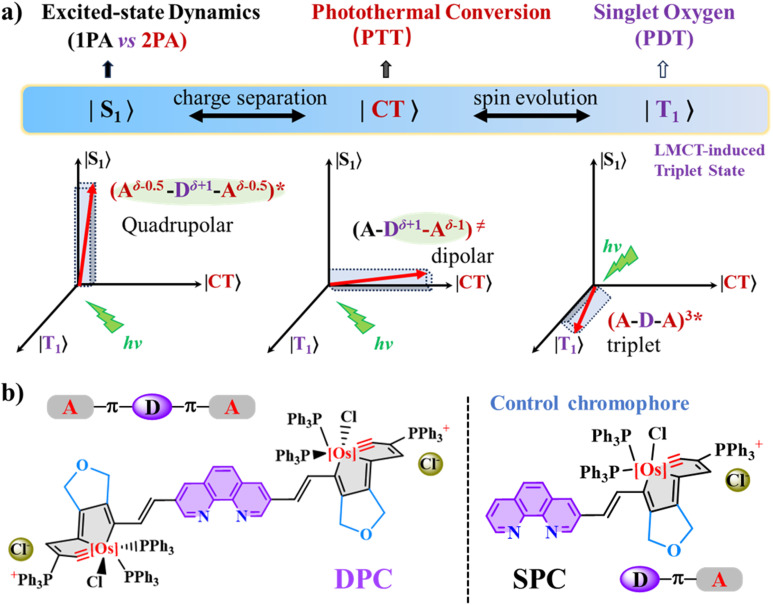
(a) Illustration of the plausible excited-state dynamics of quadrupolar metallaaromatic chromophores and potential paths for singlet oxygen generation or photothermal conversion. (b) Molecular structures of the double-phenanthroline-carbolong (DPC) and the control chromophore, single-phenanthroline-carbolong (SPC).

In principle, upon exciting the photosensitizer to the singlet excited (S_1_) state, three major photophysical pathways, including radiative transition, nonradiative relaxation, and the triplet (T_1_) state *via* intersystem crossing (ISC) – primarily occur and compete with each other.^4^ For quadrupolar and multipolar photosensitizers, the relaxed S_1_ state might transform into a charge-separated (CS) state with one-arm-bearing asymmetric excitation (Fig. 1a).^5^ On the other hand, photothermal properties correlate with nonradiative relaxation, while phototherapy utilizes the reactive oxygen species generated *via* the T_1_ state.^6^ Heavy metal atom-containing photosensitizers involve ligand ↔ metal charge transfer (LMCT or MLCT), which induces spin–orbit coupling and efficient ISC, resulting in distinct and complicated exciton coupling and excited-state relaxation dynamics. The heavy transition osmium (Os) atom facilitates significant spin–orbit coupling and efficient ISC, effectively augmenting the formation of a metastable T_1_ state.^7^ In this regard, novel metallaaromatic photosensitizers with favorable phototheranostic performances and abundant excited-state dynamics would be both interesting and challenging.

The field of metallaaromatic chemistry has recently garnered considerable attention due to the Möbius and adaptive aromaticity, diverse reactivity, excellent thermodynamic stability, and outstanding nonlinear optical (NLO) performances.^8^ Understanding their excited-state dynamics is crucial for manipulating their photophysical properties and developing related optoelectronic and phototheranostic materials. By incorporating a transition metal fragment, Xia *et al.* converted Hückel anti-aromatic into Möbius aromatic ones and established carbolong chemistry.^9^

Carbolong complexes are defined as unique metallaaromatics that contain a planar conjugated carbon chain (≥7C) chelated to a bridgehead metal by a minimum of three metal–carbon σ-bonds.^10^ Xia and co-authors have prepared metallaaromatic-loaded magnetic nanoparticles with a photothermal transduction efficiency (*η*) of 26.6% and demonstrated synergistic PTT/PDT therapies.^11^ They reported that certain metallaaromatics show exceptional PDT effects against hypoxic tumors, and most of these metal complexes do not rely on oxygen through the conventional PTT mechanism.^12^ To date, recent reports on carbolong metallaaromatics have emphasized synthesis and modification strategies for unique properties and emerging applications. Consequently, understanding the ultrafast excited-state dynamics and related optical nonlinearities of carbolong metallaaromatics has rarely been investigated.

Herein, we compared the linear photophysical behaviors and 2PA properties of dipole SPC and quadrupole DPC chromophores. The excited-state dynamics of quadrupole double-phenanthroline-carbolong (DPC) were focused on and elucidated. Its significant 2PA properties within the spectral range of 750–1100 nm were investigated using the open-aperture *Z*-scan instrument. Of particular interest are the excited-state photophysical dynamics, nonradiative relaxations, and intramolecular charge transfer (ICT) in DPC were comprehensively investigated. Ultrafast transient absorption (TA) spectra clearly revealed the excited-state dynamics of DPC, including the originally occupied Franck–Condon (FC) state, rapid ICT, and ISC processes to the T_1_ state. The long T_1_ state lifetime was evident from the stable excited-state absorption (ESA). Interestingly, singlet oxygen generation from DPC was observed when irradiated at a one-photon excitation wavelength of 638 nm and femtosecond 770 nm, considering the strong 2PA capability and large *δ*_2PA_ values. More importantly, inspired by the efficient singlet oxygen (^1^O_2_) generation and reliable photothermal conversion, the excellent phototherapeutic functions of DPC were also investigated using the 4T1 mouse breast cancer cell model. The “three-in-one” phototherapeutic effect of NIR-wavelength 2PA excitation, photodynamic therapy, and photothermal therapy in the potential photosensitizer DPC was illustrated experimentally and theoretically.

## Results and discussion

### Structural and linear optical properties

The osmapentalyne moieties of double-phenanthroline-carbolong (DPC) and single-phenanthroline-carbolong (SPC) connect electron-deficient metallaaromatic units through a rigid phenanthroline (Phen) bridge (Fig. 1b).^13^ Each Os atom is shared by two five-membered rings, featuring a coordinated metal carbyne (Os≡C) bond and significant steric hindrance from the ending carbolong framework.^14^ The DPC chromophore utilizes the same carbolong units to form its symmetrical scaffolds. The ending carbolong unit, with strong electron-withdrawing properties, and the conjugated Phen bridge result in an A-π-D-π-A structural motif. As a control chromophore, the asymmetrical SPC features an A-π-D structure. Both DPC and SPC exhibit good chemical stability and are well-soluble in common organic solvents.

**Fig. 2:**
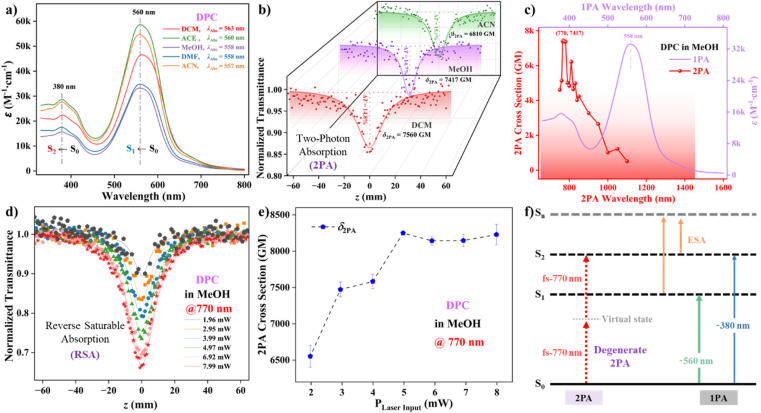
(a) UV-vis absorption spectra of DPC in different organic solvents. (b) Normalized transmittance curves and *δ*_2PA_ values of DPC in DCM, MeOH, and ACN excited at fs-770 nm. (c) Combined 1PA and 2PA spectra of DPC and related *δ*_2PA_ values in MeOH (*c* ∼ 1.0 × 10^−3^ M) excited at different fs-wavelengths ranging from 750–1100 nm. (d) Normalized transmittance diagram of DPC in MeOH with different laser input values. (e) Diagram of measured *δ*_2PA_ at fs-770 nm *vs.* input laser power at the focal plane. (f) Compared state energies diagram of the 1PA/2PA processes.

To gain insight into the ICT properties of conjugated metallaaromatic carbolong chromophores with different structures, UV-vis absorption spectra were initially performed in MeOH (Fig. S1†). Interestingly, the absorption curves of DPC and SPC exhibit similar shapes and spectral positions. The absorption peaks of the SPC chromophore appeared at 538 and 346 nm, respectively. In comparison, DPC featured a discernible absorption maximum of around 560 nm, with large molar extinction coefficients of (3.42 ∼ 5.66) × 10^4^ M^−1^·cm^−1^ (Fig. 2a and Table S1†).^14^ In contrast to the 1,10-phenanthroline fragment, which has two absorption peaks at *ca.* 230 and 263 nm, the red-shifted and broad absorption maximum of DPC can be attributed to the extended π-conjugation with the two ending metallaaromatic fragments having a strong electron-withdrawing effect.^9*c*,13*a*^ The extended π-delocalization between the d orbital of the bridgehead metal (Os) and the p orbital of the carbon chain, along with efficient ICT in the entire molecular framework, should account for the strong absorption properties.^7*b*^ Additionally, a weak solvatochromism effect, with a slight blue-shift (Δ*λ* ∼ 6 nm) was also observed along with an increase in solvent polarities, indicating the decreased energy level of the ground state solutes interacting with polar solvents (Fig. S2†). The optical bandgaps (*E*^opt^_g_) between the ground state (S_0_) and S_1_ states were found to be around 1.91 ∼ 1.94 eV in the solution states (Table S1†). The absence of aggregation interaction between neighboring DPC molecules was also demonstrated (Fig. 2a and S3†). It is important to clarify that a remarkable short-wavelength absorption band at *ca.* 380 nm with *E*^opt^_g_ ∼ 2.64 eV, probably corresponds to vibronic coupling of the S_2_ ← S_0_ transition (Table S2†). Such an assignment can also be rationalized in terms of the observed 2PA properties below. The S_1_ ← S_0_ transition dipole moments (*μ*_01_) in various organic solvents were estimated to be between 6.31 and 10.65 *D*, supporting the underlying ICT phenomenon. Unfortunately, the quadrupole DPC and dipole SPC chromophores were non-fluorescent in the investigated organic solvents. It was speculated that a rapid CT process occurred easily upon excitation, thus restraining the corresponding fluorescence emission. The substantial nonfluorescent DPC with plausible energy conversion implied alternate decay pathways for the PTT effect.

### Nonlinear 2PA properties

Owing to the strong electron-withdrawing characteristics of the carbolong units and the high electron mobility of the vinyl group, there is a large delocalization of π-electrons associated with the D–A molecular skeleton. It should be emphasized that the quadrupolar conjugated skeleton is conducive to effective electron delocalization and reduces the energy gap, thus achieving high 2PA. The degenerate 2PA performances of DPC and SPC were measured by an open-aperture *Z*-scan technique using 200 fs-long pulses.^15^ The 2PA properties of DPC in various organic solvents were measured properly, and the large *δ*_2PA_ values of DPC were approximately 7560 GM in DCM and 6810 GM in ACN, respectively (Fig. 2b). The corresponding 2PA spectra for the DPC chromophore were evaluated in the range of 750–1100 nm, which is consistent with the notable biological spectral window (650 ∼ 1450 nm) and the maximum *δ*_2PA_ value of DPC in MeOH was found to be ∼7417 GM at 770 nm (Fig. 2c, S4 and Table S3†). The *δ*_2PA_ value of SPC at 770 nm in MeOH was found to be 313 GM, with high *δ*_2PA_ values (235 ∼ 437 GM) over a wavelength range of 750–800 nm (Fig. S5†). This clarified that both molecular conjugation and ICT properties of the excited states strongly influenced the 2PA response. The observed 23-fold enhancement implied that electronic delocalization in the quadrupole A-π-D-π-A structure promoted the excited-state transition dipole moments. The characteristic reverse saturable absorption (RSA) process was demonstrated by the intense transmission valleys (Fig. 2d, S6 and S7†). Meanwhile, transmission rates at the focus decreased with increasing laser intensities, and the *δ*_2PA_ values increased appropriately (Fig. 2e).

The intensity-dependent absorption indicated that there should be higher-order nonlinear absorption, such as two-photon-induced excited-state absorption (ESA). Comparing the one-photon absorption (1PA) spectrum, the 2-fold correlation between the 2PA maximum around 770 nm and the 1PA shoulder at *ca.* 380 nm was consistent with the inherently strict selection rules for symmetric molecules and the enhanced vibronic transitions.^15*b*^ In detail, the 1PA process included S_1_ ← S_0_ (*λ*_abs_ ∼ 560 nm) and S_2_ ← S_0_ (*λ*_abs_ ∼ 380 nm) transitions. The S_1_ ← S_0_ transition (*λ*_max_ ∼ 560 nm) was 1PA-allowed, but 2PA-forbidden, whereas the strong 2PA progress allowed the S_2_ ← S_0_ (fs-770 nm) transition accompanied by ESA (Fig. 2f).^16^ According to the typical four-state model, the symmetry with a parity selection rule for the quadrupolar chromophore implied a potential S_2_ ← S_0_ transition.

### Theoretical calculations

**Fig. 3:**
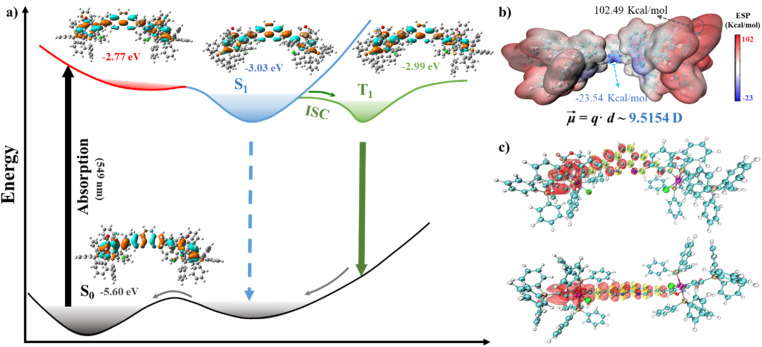
(a) Theoretical frontier molecular orbitals and energies level from DFT calculations. (b) ESP mapped and molecular dipoles of DPC in the triplet excited state T_1_. (c) Calculated spin-density surfaces of DPC with different angles at the optimized T_1_ state geometry. Notes: the red and yellow colors represent positive and negative spin densities, respectively.

To gain insights into the excited-state electronic and photophysical properties of DPC, density functional theory (DFT), and TD-DFT were performed in theoretical calculations.^7*b*,17^ According to the optimized geometries, there is partial torsion between the carbolong unit and Phen fragment due to the bulky ending groups, which well prevent intermolecular π–π interactions (Fig. S8†). Frontier molecular orbitals showed that the HOMO and LUMO electrons of DPC models were mainly distributed in the Phen and carbolong skeletons *via* the ethylene bond (Tables S4 and S5†). Meanwhile, the orbital diagrams at different energy levels were depicted to better understand the excited states process of DPC molecules (Fig. 3a). Upon photoexcitation, the first major transition of the DPC occurs at 549 nm, and the calculated HOMO–LUMO gap of the ground state S_0_ was 2.83 eV. Subsequently, there was a rapid transfer from the unstable LUMO of S_0_ to the S_1_ state, which then formed the charge-separated T_1_ species *via* ISC. Due to the low energy gap between the S_1_ and T_1_ states (Δ*E*_ST_), the excited DPC system efficiently achieved the ISC process to form the T_1_ species (^3^MLCT), thereby promoting the production of reactive oxygen species (ROS) through electron transfer to molecular oxygen. Moreover, the energies of the stronger transitions in the computed UV-vis absorption spectra aligned well with the experimental values for DPC, with only a modest overestimation of transition energies (Fig. S9†). The transition at 549 nm was predominantly determined to be of a ^1^MLCT character, consisting of a mixed HOMO → LUMO (88.5%) and HOMO−1 → LUMO+1 (9.3%), where the additional electron density was localized in the Phen unit. For the second major transition at 386 nm, the character of HOMO−6 → LUMO exhibited significant charge transfer from carbolong to Phen, revealing that ^1^MLCT transitions occurred at higher energy.^18^

The electrostatic potential (ESP) on the van der Waals surface was analyzed by quantitative molecular surface to locate ESP maxima and minima (Fig. 3b). The positive ESP covered most of DPC, while the electron-rich region is mainly located around the N atom of phenanthroline and the Cl atom of the carbolong. Notably, the asymmetric T_1_ structure exhibited a larger dipole moment (9.51 *D*) due to a sufficiently large charge transfer, which is consistent with the optimized geometries.^19^ The spin density surfaces revealed that the T_1_ state was primarily located on the side of the DPC chromophore (Fig. 3c), confirming the ^3^MLCT character of the T_1_ excited state. The spin density was dispersed across the Os center and carbolong ligand and could be safely designated as ^3^MLCT.^20^

### Nanosecond ultrafast transient absorption (ns-TA) and singlet oxygen measurements

**Fig. 4:**
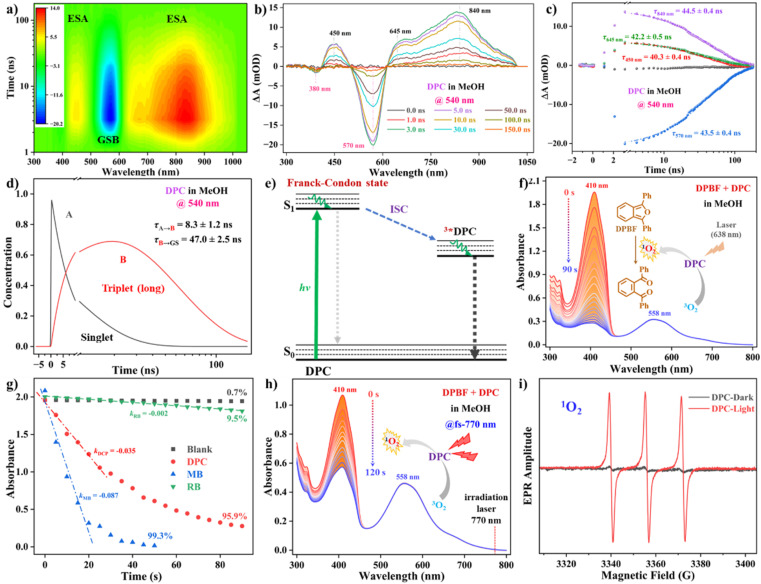
Related 2D contour map (a) and the ns-TA spectra (b) of DPC excited at 540 nm in MeOH. (c) Overlay plots of specific kinetic traces with corresponding curves at various wavelengths. (d) Species-associated component spectra. (e) Schematic diagram of the photoexcitation, ISC, and relaxation processes in the nanosecond time scale. (f) Time-dependent UV-vis absorption spectral changes of DPC/DPBF during the irradiation time (*t*) of 90 s. Notes: 638 nm laser, 0.5 W cm^−2^. (g) Plots of absorbance *vs.* laser irradiation times with different photosensitizers and DPBF present. (h) Time-dependent UV-vis absorption spectral changes of DPC/DPBF during the irradiation time of 120 s. Notes: 770 nm fs-laser, 0.2 W cm^−2^. (i) EPR spectra to detect ^1^O_2_ generated by DPC with/without green light irradiation using TEMP as a spin-trap agent.

Spectroscopy is among the important methods to study the photoinduction kinetics of conjugated chromophores and explore their photophysical processes.^21^ We conducted ns-TA measurements to investigate and elucidate the T_1_ excited state properties first. Upon photoexcitation of DPC at 540 nm, negative ground-state bleaching (GSB) signals centered at 380 and 570 nm were observed, consistent with the corresponding steady-state absorption spectrum (Fig. 4a). A broad positive ESA contribution assigned for the S_*n*_ ← S_1_ transitions was found in the 615 ∼ 1020 nm spectral region, with three maxima centered at 450, 645, and 840 nm (Fig. 4b). The ns-TA spectral intensity declined without a noticeable spectral shift in the whole tested spectral region, from the perspective of a longer time decay of 150 ns. A first order kinetic fit of the characteristic wavelengths yielded lifetimes of 44.0 ns for DPC in MeOH (Fig. 4c). It is noteworthy that the similar kinetic traces of ESA bands at 645 and 840 nm clearly indicated that these two wavelengths were related to the newly formed CS state and T_1_ species ^3^MLCT. The dynamics of ns-TA spectra were fitted with a sequential kinetic model and global kinetic fitting (Fig. 4d and e, S10†). It observed that the transition to the T_1_ state occurred at approximately 8.3 ns in MeOH. It was hypothesized that the phototransient formed upon excitation in the nanosecond timescale was probably an asymmetrical charge separation state before the T_1_ state. The corresponding kinetic characteristics of DPC decaying from the T_1_ state back to the S_0_ state were initially estimated to be 47.0 ns. The Os atom's strong spin–orbit coupling could induce efficient ISC to the T_1_ state.^22^ The long-lived T_1_ state illustrated considerable stability against oxygen and recombined over 120 ns to the ground state (Fig. S11–S13 and Tables S6, S7†).

To gain more insight into the efficient ISC and verify that the observed long-lived components stemmed from the T_1_ state in DPC, its singlet ^1^O_2_ generation capabilities were assessed under light irradiation using 1,3-diphenylisobenzofuran (DPBF) as an indicator.^23^ Upon 638 nm laser irradiation for 90 s, the absorption of DPC/DPBF in MeOH at 410 nm decreased rapidly, confirming the efficient production of ^1^O_2_ (Fig. 4f). When illuminating the mixtures of DPBF/MB and DPBF/DPC, the absorption maxima of DPBF sharply decreased by 99.3% and 95.9% (Fig. 4g, S14 and S15†), respectively, demonstrating a more effective ^1^O_2_ generation capacity of DPC, plausibly through the type II photochemical mechanism. The ^1^O_2_ yield (*Φ*_Δ_) of DPC in MeOH was characterized to be ∼8.4% using methylene blue as a standard (*Φ*_Δ_ = 52% in MeOH) (Table S8†).^1*b*^ Notably, considering the large 2PA capabilities, fs-770 nm laser irradiation at DPC/DPBF for 120 s induced an obvious 46.6% reduction of DPBF at 410 nm, in contrast to the values of 10.8% and 11.3% for DPBF/RB and DPBF/MB, respectively (Fig. 4h, S16 and S17†). Furthermore, electron paramagnetic resonance (EPR) spectroscopy was implemented to confirm the generation of ^1^O_2_ utilizing 2,2,6,6-tetramethylpiperidine (TEMP) (Fig. 4i). Under irradiation, EPR spectra of the DPC and TEMP mixing solution showed a trilinear signal with an intensity of 1 : 1 : 1 between 3334 ∼ 3378 *G*, which supported the generation of ^1^O_2_.^12,24^

### Femtosecond ultrafast transient absorption (fs-TA) measurements

**Fig. 5:**
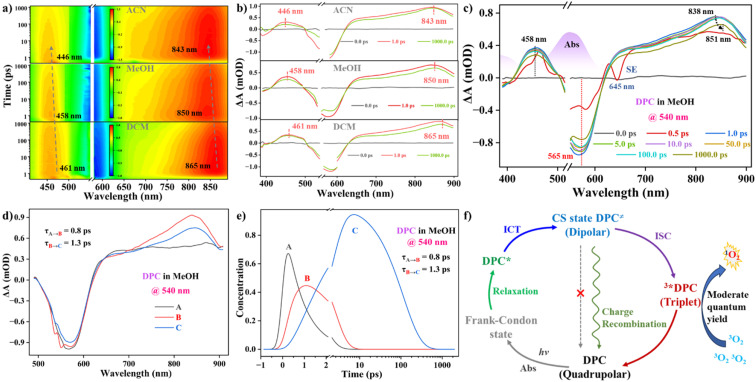
Related 2D contour map (a) and fs-TA spectra (b) of DPC in ACN, MeOH, and DCM. (c) 3D fs-TA spectral shift of the excited DPC in MeOH within the initial 1000 ps. Species-associated spectra (d), component spectra (e), and a plausible kinetic model (f) related to the fs-TA spectra and the generation of ^1^O_2_.

We employed fs-TA spectra to further investigate the polarity-dependent ICT nature of DPC in the excited-state dynamics.^2*b*^ All spectra exhibited complicated changes in broadband shape and amplitude occurring over the instrument time scale of 6.0 ns. Upon photoexcitation, the immediate formation of a negative GSB band around 565 nm and strong positive and broad ESA bands peaked from 623 nm to 895 nm in different solvents was properly assigned (Fig. 5a and b). This indicated that the photogenerated excitons in all higher excited states could undergo a fast internal conversion process to S_1_.^25^ With increasing solvent polarity, blue-shifted ESA signals indicated progress in ICT on a short time scale. No obvious recovery of the GSB and ESA bands within 6.0 ns supported the long excited-state lifetime of DPC in the nanosecond time regime. The depopulation of the S_1_ state provided unambiguous evidence for efficient CT processes and the singlet–triplet transition.

Interestingly, analyzing the fs-TA delays within the initial few picoseconds, the GSB band showed up at the longer 590 nm upon freezing to the FC state. The initially occupied FC state decayed rapidly as the ESA features evolved over time, displaying characteristic spectral hallmarks of the various excited states of DPC. The GSB peak then blue-shifted to 565 nm in the longer time delay (Fig. 5c). Additionally, we could observe the stimulated emission (SE) trace at ∼645 nm, which quickly overlapped with the strong and broadening ESA signals above 615 nm, supporting the fast decay from the initial FC to CT state and the low radiative rate. Notably, the early ESA bands appeared in a broad spectral region and peaked at 838 nm. Furthermore, the intense ESA bands red-shifted to 851 nm, corresponding to the early-time CT state. Based on a comprehensive inspection of the fs-TA spectra employing multiwavelength and global analysis, four transient species were necessary for a satisfactory match (Fig. S18†). One of these, following the FC state filled by ultrafast optical excitation, was ascribed to the excited DPC*, accompanied by solvent reorganization and structural relaxation, which happened on a short time scale (*τ*_A→B_ ∼ 0.8 ps). After a total lifetime of approximately 1.3 ps (*τ*_B→C_) for the quick ICT process, the primary CS species, DPC^≠^, was formed properly, and further asymmetric deformation in the A-π-D-π-A moiety occurred (Fig. 5d and e). Combined with ns-TA analysis, the CS species DPC^≠^ decayed mainly in two ways: *via* a nonradiative pathway through charge recombination (CR) to the S_0_ state and through photoconversion into the T_1_ state through ISC. The fourth transient species is likely the populated T_1_ state after a fast ISC process from S_1_, which then decayed to the S_0_ state within 47.0 ns. A kinetic model of the excited-state processes illustrated the observed excited-state dynamics according to the energy level diagram, highlighting the two competitive energy dissipation pathways (ISC and CR) (Fig. 5f).

### Photothermal conversion studies and phototoxicity *in vitro*

**Fig. 6:**
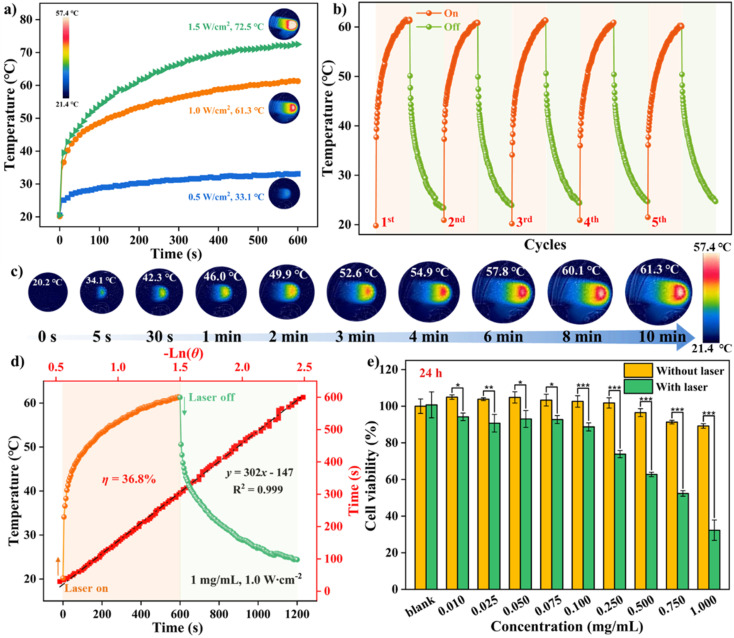
(a) Photothermal heating curves of DPC (660 μL, 1.00 mg mL^−1^) following exposure to 638 nm laser at various power densities. (b) Five photothermal heating curves for DPC under laser on-off irradiation. (c) Infrared thermographies of DPC in a water–DMSO solution (9/1, v/v, 1.00 mg mL^−1^, 660 μL) under laser irradiation at various time points. (d) Temperature rise/fall curves and the cooling time *vs.* –ln(*θ*) plot. Notes: *θ* represents the driving force temperature for DPC. (e) Cell viability of 4T1 cells incubated with DPC at varying concentrations (0.01 ∼ 1.00 mg mL^−1^) for 24 h with or without laser irradiation. Notes: 638 nm laser, 1.0 W cm^−2^.

Further applications of DPC in photothermal conversion were explored. The temperature of a DPC solution in water–DMSO (9/1, v/v, 660 μL, 1.00 mg mL^−1^) increased upon continuous irradiation with a 638 nm laser. When the laser power reached 1.0 and 1.5 W cm^−2^, the solution temperatures rapidly rose to 61.3 °C and 72.5 °C (Fig. 6a and S19†), respectively.

Moreover, no substantial deterioration of photothermal conversion performance was observed even after five irradiation cycles (Fig. 6b), illustrating the satisfactory photostability and thermostability of DPC. The high-contrast infrared thermographs, along with increased irradiation time up to 10 min, confirmed the outstanding photothermal effect (Fig. 6c). As illustrated in the heating and cooling curves under laser on-off switch irradiation at 1.0 W cm^−2^, the solution temperature quickly increased from 20.2 °C to 49.9 °C within 120 s, then gradually rose to 61.3 °C within 10 min. The relationship of cooling time *vs.* the generated negative natural logarithm {−ln(*θ*)} indicated that the photothermal conversion efficiency (*η*) of the present system was estimated to be ∼36.8%, with the heat transfer time-constant (*τ*_s_) determined to be 302 s (Fig. 6d).

Recently, “all-in-one” photosensitizers featuring multiple photosensitive roles have shown promise for cancer therapy and pose challenges for achieving high-performance therapeutic efficacy. Inspired by the efficient ^1^O_2_ generation and reliable photothermal conversion of DPC, the phototherapeutic functions were evaluated using a 4T1 model system with the CCK-8 method. Initially, 4T1 cells treated with specific DPC concentrations ranging from 0.01 to 1.00 mg mL^−1^ retained high survival rates of over 90% under dark conditions, indicating low dark cytotoxicity of DPC even within 48 h of incubation (Fig. S20†). To further investigate the phototherapy effect, different irradiation times ranging from 0–15 min were assessed, with 5 min being optimized (Fig. S21†). To our delight, laser irradiation alone exhibited negligible cytotoxicity (Fig. S22†). DPC showed significantly increased cytotoxicity when irradiated at 638 nm, exhibiting superior PTT capability, especially when its concentration exceeded 0.25 mg mL^−1^ (Fig. 6e). More importantly, *in vitro* experiments demonstrated the PTT/PDT synergistic effect of DPC in 4T1 cells. The half maximal inhibitory concentration (IC_50_) value of DPC after 24 h of incubation was determined to be 0.687 mg mL^−1^, respectively (Fig. S23†). These results demonstrate the “three-in-one” phototherapeutic effect of the NIR-wavelength 2PA excitation, PDT, and PTT in the potential photosensitizer DPC.

## Conclusions

The photophysical properties of a phenanthroline-carbolong photosensitizer (DPC) have been comparatively investigated. Significant nonradiative relaxations and DFT calculations indicated that a CS state was produced upon excitation. Outstanding *δ*_2PA_ values of up to 7417 GM in MeOH at 770 nm were assigned to the higher excited state (S_2_ ← S_0_), with presumably significant contributions from charge-separated ESA. The ns/fs-TA spectra of DPC revealed the plausible transition dynamics from the initially populated FC to the primary CS state. Meanwhile, competing with the ISC process, part of the DPC^≠^ charge recombined to the ground state, displaying a high photothermal conversion efficacy of around 36.8%. Furthermore, the ^1^O_2_ yield of DPC in MeOH was found to be ∼8.4% under 638 nm, and DPC with a large *δ*_2PA_ simultaneously produced ^1^O_2_ in the biological spectral window (650 ∼ 1450 nm). *In vitro* experiments demonstrated the excellent synergistic effect of PTT and PDT using DPC in 4T1 cells, which realized a “three-in-one” phototherapeutic strategy with NIR-wavelength 2PA excitation. These results not only shed light on the fundamental excited state dynamics of the metallaaromatic chromophores but also provide essential guidelines for designing potential nonlinear and phototheranostics materials.

## Data availability

The data that support the findings of this article are available from the corresponding author upon reasonable request.

## Author contributions

The manuscript was written through the contributions of all authors. H. Chang performed the majority of the experimental work and data analyses and wrote the original draft. J. Feng and X.-A. Liu participated in the data analysis. R. Miao and T. Liu supervised the experimental work, analyzed the data, and revised the manuscript. L. Ding and Y. Fang supervised the project and revised the manuscript.

## Conflicts of interest

There are no conflicts to declare.

## Supplementary Material

SC-OLF-D5SC00013K-s001
