# Supplementary material for: Ultrafast excited-state dynamics and “three-in-one” phototheranostic properties of a phenanthroline-carbolong photosensitizer
Source: Chem Sci. 2025 Mar 14;16(17):7424–32. doi: 10.1039/d5sc00013k (PMC11948273; doi:10.1039/d5sc00013k)
Supplement: SC-016-D5SC00013K-s001 [file SC-016-D5SC00013K-s001.pdf]

Supplementary Information (SI) for Chemical Science.
This journal is © The Royal Society of Chemistry 2025

Electronic Supplementary Information

for

Ultrafast excited-state dynamics and "three-in-one" phototheranostic properties of a phenanthroline-carbolong photosensitizer

Haixia Chang, Jiang Feng, Xin-Ao Liu, Rong Miao,* Taihong Liu,* Liping Ding and Yu Fang

Institute of New Concept Sensors and Molecular Materials, Key Laboratory of Applied Surface and Colloid Chemistry of Ministry of Education, School of Chemistry and Chemical Engineering, Shaanxi Normal University, Xi'an 710119, People's Republic of China

Corresponding authors: liuth121@snnu.edu.cn (T. Liu); miaorong2015@snnu.edu.cn (R. Miao)

Content

1. Experimental section …………………………………………………………………………………...……… S3

2. Supplementary optical properties ……………………………………………….…………………….…………..... S4

3. Z-scan measurement and two-photon absorption (2PA) ……………..…………………………..…………………... S5

4. Quantum chemical calculations ……………..…………………….. S8

5. Nanosecond transient absorption (ns-TA) spectroscopy …………………………………………….……..........…... S10

6. Singlet oxygen quantum yield (Φ_Δ) …………………..………….……………………….....….………….......... S11

7. Femtosecond transient absorption (fs-TA) spectroscopy ……………………………………………….……………… S13

8. Photothermal conversion studies ……………………………..………………………………….…………..…. S14

References ……………………………………….……….....……………………………..……….………...……… S17

1. Experimental section

1.1. Reagents and characterization instruments

The double-phenanthroline-carbolong chromophore DPC (95%) covalently linked phenanthroline with two ending 7C-carbolong units, and 2,2,6,6-tetramethylpiperidine (TEMP, 98%) were commercially obtained from J&K Scientific.[1] Centrosymmetric DPC featured an acceptor-π-donor-π-acceptor (A-π-D-π-A) structural motif with quadrupolar characteristics. The control chromophore single-phenanthroline-carbolong SPC was provided kindly by Dr. Shiyan Chen in Xia's group (Southern University of Science and Technology).[1] Meanwhile, 1,10-phenanthroline (Phen, 98%) was obtained from Bide Pharmatech Ltd. Dichloromethane (DCM), *N*,*N*-dimethylformamide (DMF), acetone (ACE), acetonitrile (ACN), methanol (MeOH), and other reagents were commercially available without further purification unless otherwise noted. Steady-state UV-vis absorption spectra were recorded using a Shimadzu UV-2600 spectrometer in a 1.0 cm path-length quartz cuvette. The corresponding full width at half maxima (FWHM) were calculated properly. Steady-state fluorescence emission spectra were checked using a fluorescence spectrometer (Edinburgh FLS1000) with a 450 W xenon arc lamp as the light source at room temperature. Electron paramagnetic resonance (EPR) spectra were performed using a Bruker E500 spectrometer.

1.2. Cell culture and in vitro cytotoxicity

The 4T1 cells were cultured in PPMI 1640 medium with 10% fetal bovine serum at 37 °C in a humidified atmosphere containing 5% CO_2. Then, 4T1 cells were seeded into 96-well plates overnight at a density of 8.0×10^3 cells/well. The cell medium was replaced with fresh medium in the presence of DPC with different concentrations (0.01 ~ 1.00 mg/mL) and incubated for 24 h or 48 h, followed by determining the cell viability with CCK-8 method.

1.3. Calculation details of the transition dipole moments and optical bandgaps

Transition dipole moments ($\Delta\mu$) between the ground state (S_0) and the excited states were obtained by integration of the main low energy absorption bands of chromophore DPC and calculated as follows:

$$\Delta\mu = 0.09584 \times \sqrt{\int \frac{\varepsilon(v)}{v^{max}} dv}$$

Where $\varepsilon(v)$ is the molar extinction coefficient of transition, v^{max} is the maximum absorption wavelength in cm^{-1}. The transition dipole moments μ_{01} and μ_{02} were denoted for S_0 to the first excited state (S_1) and to the second excited state (S_2), respectively.

The optical bandgap (E_g^{opt}) is obtained according to the following equation, $E_g^{opt}(eV) = 1240/\lambda_{edge}$, where λ_{edge} is the onset value of absorption spectrum in the long wavelength direction.

2. Supplementary optical properties

2.1. Compared UV-vis absorptions of DPC and the control chromophores in kinds of organic solvents

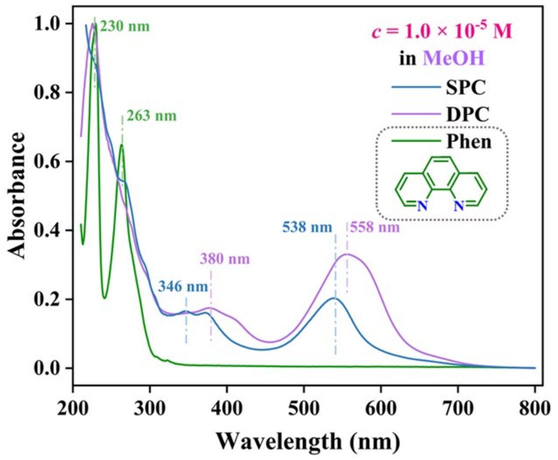

Fig. S1. Combined UV-vis spectra of the phenanthroline-carbolong chromophrores DPC, SPC, and 1,10-phenanthroline (Phen) in MeOH. *Notes*: $c \sim 1.0 \times 10^{-5}$ M.

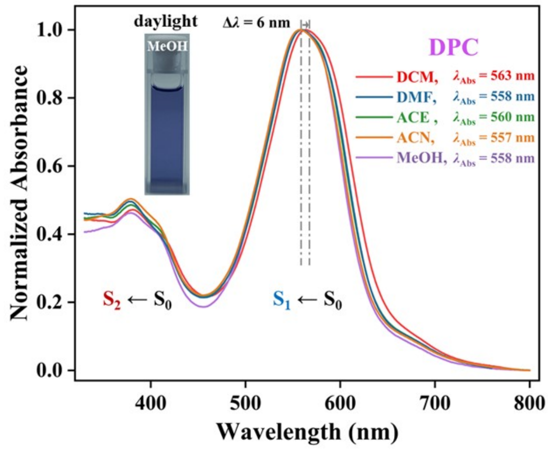

Fig. S2. Normalized UV-vis spectra of DPC in kinds of organic solvents at room temperature. *Notes*: Inset showed the photograph of DPC in MeOH under daylight.

Table S1. Summarized optical properties of DPC for the $S_1 \leftarrow S_0$ transition.

Solv.	Δf	$E_T(30)$	λ^{0-1}_{Abs}	FWHM	E^{0-1}_{g}	$\varepsilon_{0\text{-}1}$	μ_{01}	σ
		kcal·mol^{-1}	nm	nm	eV	M^{-1}·cm^{-1}	D	cm^2
**DCM**	0.217	41.1	563	107	1.91	4.62×10^4	6.31	1.76×10^{-16}
**DMF**	0.274	43.8	558	105	1.91	3.61×10^4	8.59	1.38×10^{-16}
**ACE**	0.287	42.2	560	103	1.93	5.66×10^4	10.04	2.16×10^{-16}
**ACN**	0.304	46.0	557	102	1.94	5.42×10^4	10.65	2.07×10^{-16}
**MeOH**	0.308	55.5	558	100	1.94	3.42×10^4	8.22	1.31×10^{-16}

Table S2. Summarized optical properties of DPC for the $S_2 \leftarrow S_0$ transition.

Solv.	Δf	$E_T(30)$	λ_{Abs}^{0-2}	FWHM	E_g^{0-2}	$\varepsilon_{0\text{-}2}$	μ_{02}	σ
		kcal·mol^{-1}	nm	nm	eV	M^{-1}·cm^{-1}	D	cm^2
**DCM**	0.217	41.1	381	97	2.56	2.24×10^4	2.78	8.56×10^{-17}
**DMF**	0.274	43.8	378	82	2.56	1.75×10^4	2.51	6.69×10^{-17}
**ACE**	0.287	42.2	379	85	2.62	2.87×10^4	3.22	1.10×10^{-16}
**ACN**	0.304	46.0	378	84	2.66	2.76×10^4	3.09	1.05×10^{-16}
**MeOH**	0.308	55.5	380	85	2.66	1.56×10^4	2.77	5.96×10^{-17}

2.2. Concentration effect of DPC in the selected organic solvents

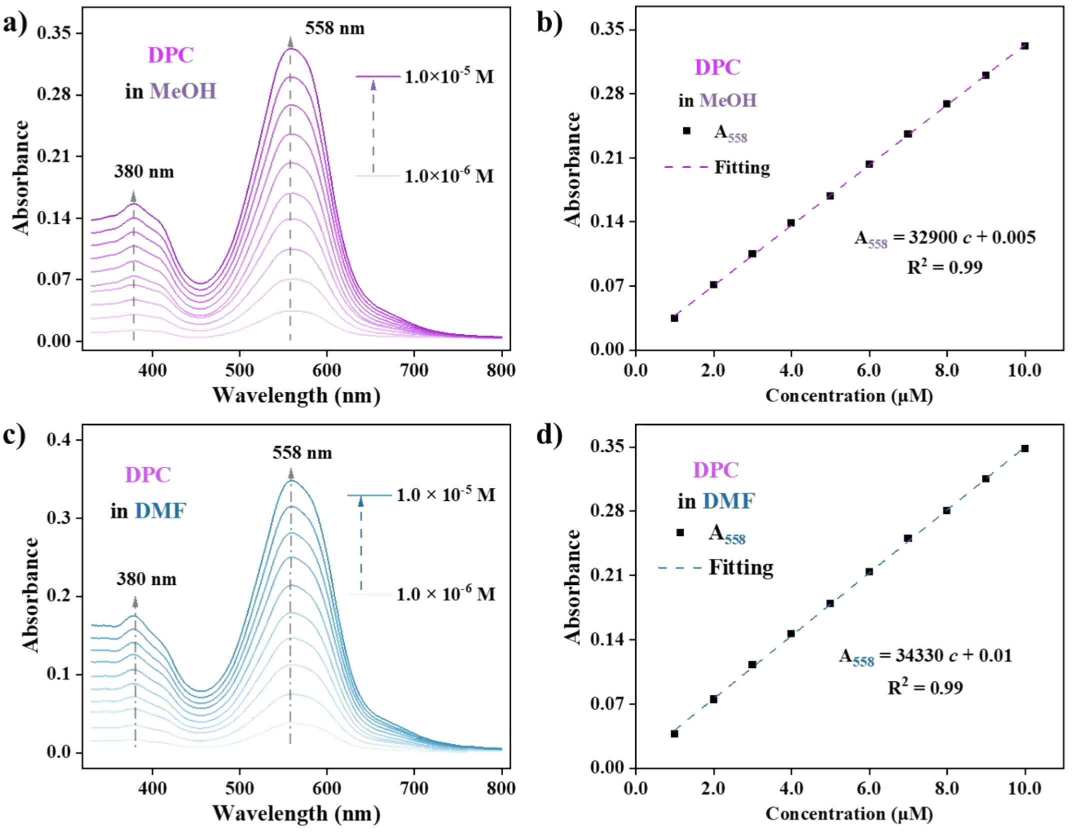

Fig. S3. Concentration effect of DPC ($c = 1.0 \times 10^{-6} \sim 1.0 \times 10^{-5}$ M) in MeOH (a) and DMF (c). Linear correlations of the absorbance intensity of DPC with the concentration of DPC in MeOH (b) and DMF (d).

3. Z-scan measurements and two-photon absorption (2PA)

The experimental setup for femtosecond Z-scan absorption measurements in the present work are outlined in Scheme S1.[2,3] In the open aperture Z-scan technique developed by Sheik-Bahae *et al*,[4] the 2PA cross section (δ_{2PA}) values in the 750 ~ 1100 nm spectral region were determined by translating the sample through the focal plane of a

focused Gaussian beam, while transmittance changes in the far field intensity were monitored. In detail, the laser source is a mode-locked Yb:KGW laser (Light Conversion, Pharos) with a pulse width of 200 fs and a repetition rate of 10 kHz. The calculated Rayleigh length z_0 was greater than the thickness of sample cuvette (1.0 mm), an essential requirement for Z-scan experiments. Using the open aperture Z-scan configuration, the nonlinear absorption coefficient (β) of the sample was measured. Referenced to the known 2PA cross section of Rhodamine B (~ 53 GM at 770 nm) listed in the literatures, each measurement is repeated multiple times and averaged to retrieve the NLO coefficients.[5,6] The uncertainties in the NLO coefficients and δ_{2PA} are estimated to be commonly ~ 15% arising predominantly from a) errors in the estimation of beam waist (at focus) and thereby leading to errors in the peak intensities, which is a crucial parameter, b) fluctuation in the input laser power, and c) fitting errors, *etc*. The unit of δ_{2PA} is defined as 1 GM ≡ 10^{-50} $cm^4 \cdot s \cdot photon^{-1} \cdot molecule^{-1}$. Pure solvent samples under the same experimental conditions showed no significant results.

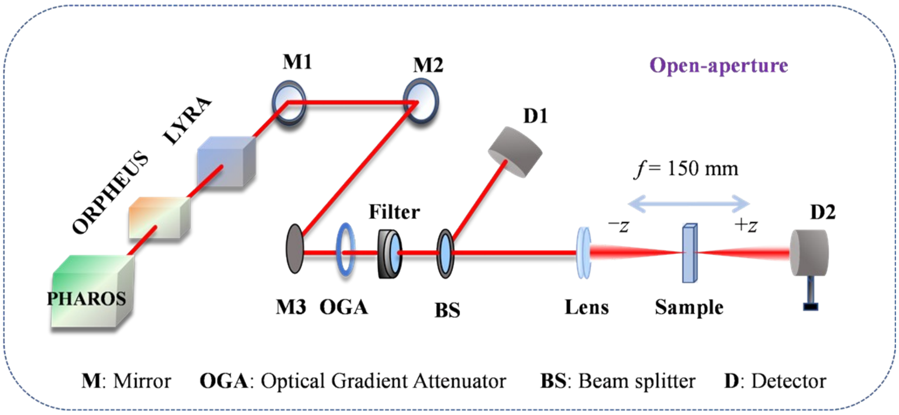

Scheme S1. Schematic diagram of femtosecond open-aperture Z-scan set-up in 2PA measurements.

Nonlinear optical parameters can be obtained by curve fitting to the observed open aperture traces with the following equations. δ_{2PA} values are obtained from experimental data by fitting Z-scan curves (S1), where q_0 is a parameter related to the coefficient β, the input intensity I_0 at the focal point in the absence of sample, and the effective sample length L_{eff}. z_0 is the Rayleigh range. w_0 is the beam waist at the focal point. z is the sample position. δ_{2PA} is calculated from β through equation S5, where N_A is the Avogadro's number, c is the sample concentration, and $h\nu$ is related to the excited photon energy.

$$\Delta T(z) = \frac{q_0(0)}{2\sqrt{2}} \frac{1}{1 + (z/z_0)^2} \quad \text{(S1)}$$

$$q_0(0) = \beta I_0 L_{eff} \quad \text{(S2)}$$

$$L_{eff} = (1 - e^{-\alpha_0 L})/\alpha_0 \quad \text{(S3)}$$

$$z_0 = \frac{n\pi\omega_0^2}{\lambda} \quad \text{(S4)}$$

$$\delta_{2PA} = \frac{1000 \times h\nu\beta}{N_A c} \quad \text{(S5)}$$

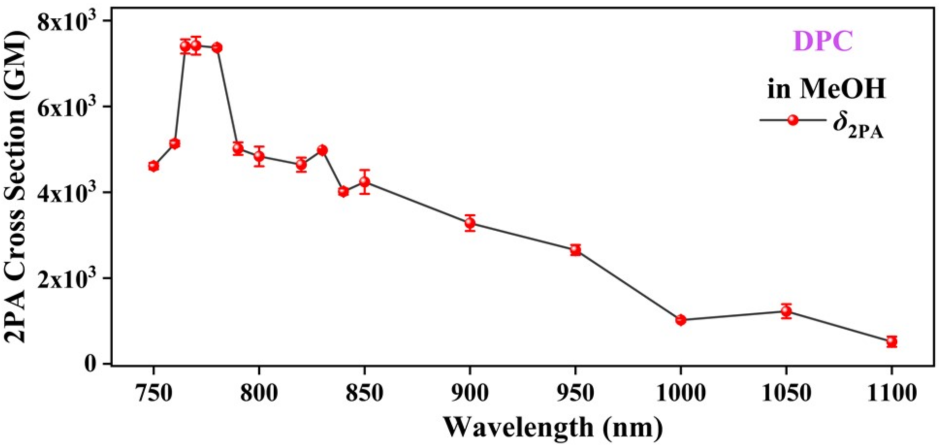

Fig. S4. 2PA cross section (δ_{2PA}) of DPC in MeOH (c ~ 1.0 × 10^{-3} M) excited with different femtosecond wavelengths ranging from 750 nm to 1100 nm.

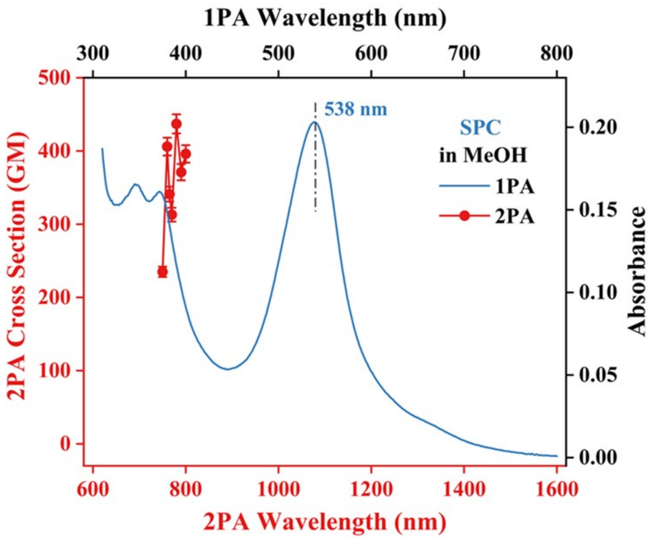

Fig. S5. Combined 1PA and 2PA spectra of the control chromophore SPC and related δ_{2PA} values in MeOH (c ~ 1.0 × 10^{-2} M) excited at different femtosecond wavelengths ranging from 750 ~ 800 nm.

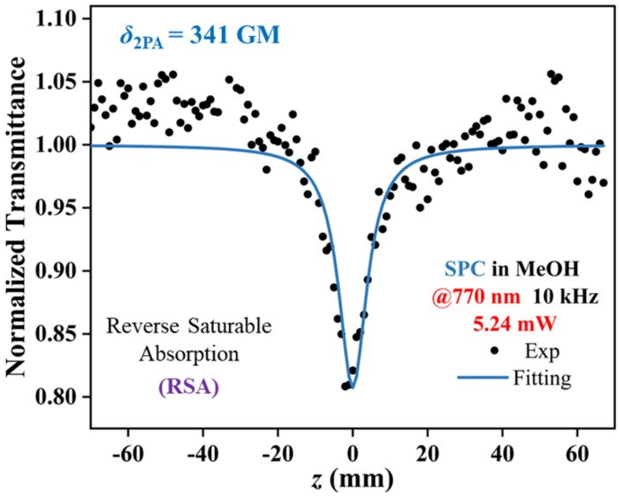

Fig. S6. Normalized transmittance curves and δ_{2PA} value of SPC in MeOH measured at fs-770 nm.

Table S3. Compared δ_{2PA} values between DPC and SPC in MeOH excited with different femtosecond wavelengths.

λ/nm	δ_{2PA}/GM		λ/nm	δ_{2PA}/GM	
	DPC	SPC		DPC	SPC
750	4610	235	830	4980	---
760	5137	406	840	4017	---
765	7400	341	850	4240	---
770	7417	313	900	3280	---
780	7370	437	950	2653	---
790	5017	371	1000	1021	---
800	4837	396	1050	1227	---
820	4643	---	1100	516	---

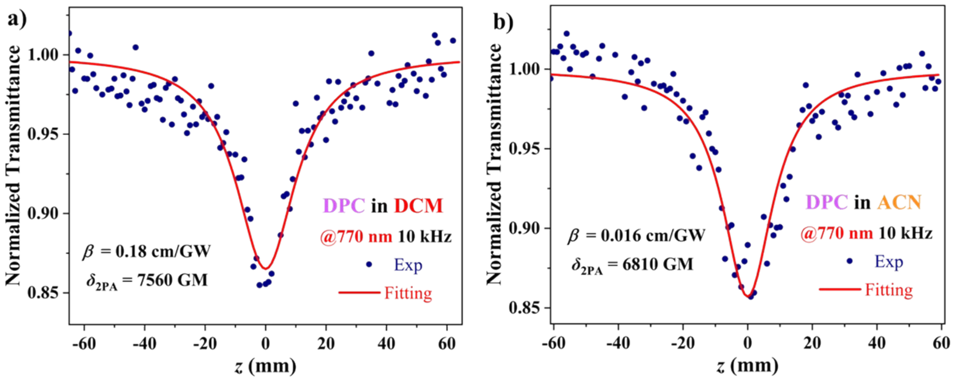

Fig. S7. Normalized transmittance curves and δ_{2PA} values of DPC in DCM (a) and ACN (b) at fs-770 nm.

4. Quantum chemical calculations

To better understand the structure-property correlation, theoretical calculations of the carbolong chromophore

DPC were carried out by the Gaussian 16 software. Geometry optimizations and energy calculations were performed at the level of (TD-)PBE0/SDD[7] for the metal ion Os and PBE0/6-31G(d) [8,9] for the other atoms in MeOH. The wave functional analysis was calculated by the Multiwfn program and the isosurface maps were rendered by the VMD 1.9.3 software.[10]

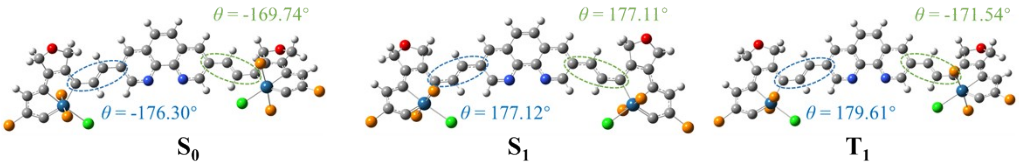

Fig. S8. Optimized geometries and dihedral angles of DPC in the ground state S_0, the singlet excited state S_1, and the triplet excited state T_1. The phenyl units on the phosphorus atoms were omitted for clarity.

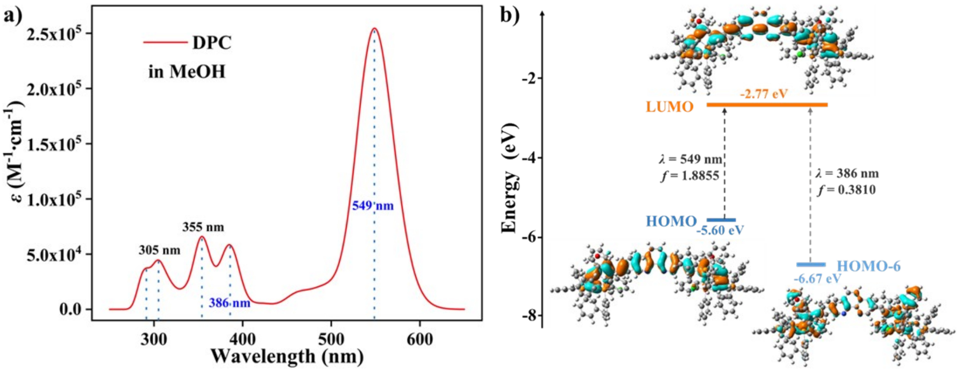

Fig. S9. Fitted UV-vis spectrum (a) and frontier molecular orbitals and energies (eV) from DFT calculations (b) of DPC in MeOH by Gaussian.

Table S4. Excited states, symmetry, transitions, wavelengths and oscillator strength of DPC.

Excited State	Symmetry	Transitions	Energy (eV)	Wavelength (nm)	Osc. Strength (*f*)
S_0	Singlet-A	H → L (88.5%)<br>H-1 → L+1 (9.3%)	2.2590	548.85	1.8855
S_1	Singlet-A	H → L (94.7%)<br>H-1 → L+1 (4.3%)	1.8918	655.36	2.2751
T_1	Triplet-A	H-1 → L (4.8%)<br>H-1 → L+1 (2.8%)<br>H → L (86.8%)<br>H → L+1 (10.7%)<br>H ← L (8.6%)	0.8187	1514.35	0

Table S5. Frontier molecular orbitals and energy levels of DPC in the ground state S_0, the singlet excited state S_1,

and the triplet excited state T_1.

Excited State	HOMO-1	HOMO	LUMO	LUMO+1
S_0	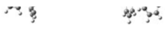 -5.83 eV	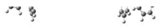 -5.60 eV	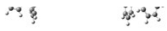 -2.77 eV	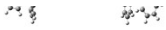 -2.48 eV
S_1	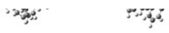 -5.73 eV	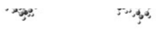 -5.42 eV	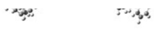 -3.03 eV	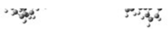 -2.57 eV
T_1	---	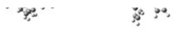 -5.38 eV	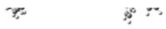 -2.99 eV	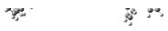 -2.53 eV

5. Nanosecond transient absorption (ns-TA) spectroscopy

ns-TA spectra were measured using a Nano-TA100 spectrometer from Time-tech Spectra. Probe beam was generated by a nanosecond Nd:YAG laser (Disco UV, Leukos, 420 nm - 2400 nm). Pump beam was generated by a femtosecond PHAROS laser system with Light Conversion (1030 nm, < 190 fs, 200 μJ/pulse, and 100 kHz repetition rate). PHAROS was set up to generate 560 nm or 540 nm excitation pulse. The liquid samples (2.0 mm quartz cell) were settled on the platform at the intersection of the probe beam and the excitation pulse. All the samples are optically diluted at the laser excitation wavelength. The excited state lifetimes were obtained by kinetic analysis of the transient absorption. All the spectra were measured at room temperature if no further notification. Furthermore, after deaerating the samples with N_2 for *ca.* 20 min before measurement, the intrinsic parameters for the deoxygenated DPC were obtained by fitting the experimental decay traces.

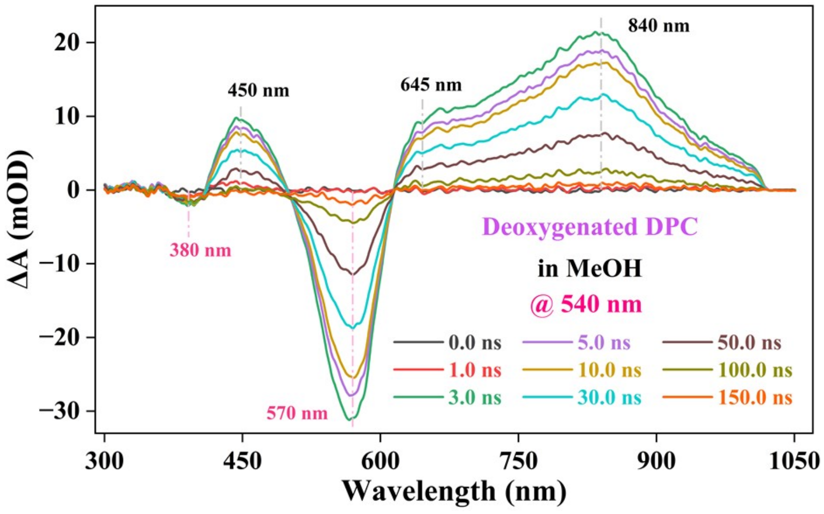

Fig. S10. ns-TA spectra of deoxygenated DPC excited at 540 nm.

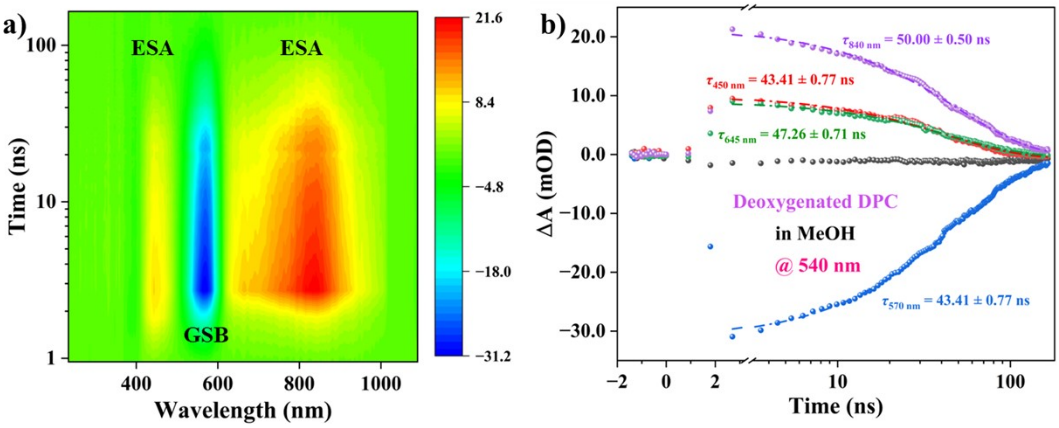

Fig. S11. a) Related 2D contour map of the ns-TA spectra of deoxygenated DPC in MeOH. b) Plots of selected kinetic traces superimposed with matching curves at different wavelengths.

Table S6. Summarized dynamic parameters by fitting ns-TA kinetic attenuation curves.

Compounds	τ_{570} / ns	τ_{450} / ns	τ_{645} / ns	τ_{840} / ns
Deoxygenated DPC	43.41 ± 0.77	43.41 ± 0.77	47.26 ± 0.71	50.00 ± 0.50
DPC	43.46 ± 0.44	40.32 ± 0.36	42.23 ± 0.53	44.48 ± 0.40

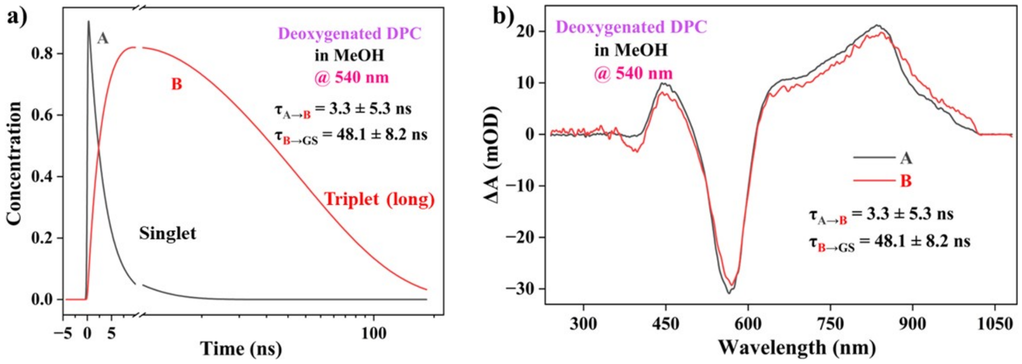

Fig. S12. Species-associated spectra plots (a) and kinetic model plots (b) related to the ns-TA spectra of deoxygenated DPC.

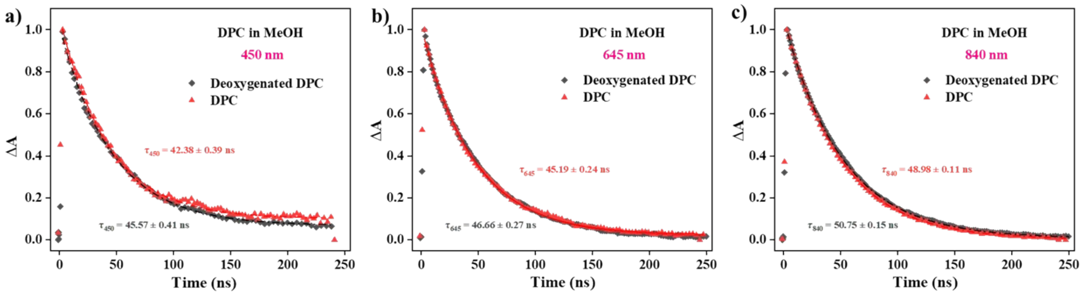

Fig. S13. Kinetic attenuation curves related to the ns-TA spectra of deoxygenated and undeoxygenated DPC in MeOH at 450 nm (a), 645 nm (b) and 840 nm (c).

Table S7. Summarized dynamic parameters by fitting ns-TA kinetic attenuation curves.

Compounds	τ_{450} / ns	τ_{645} / ns	τ_{840} / ns
Deoxygenated DPC	45.57 ± 0.41	46.66 ± 0.27	50.75 ± 0.15
DPC	42.38 ± 0.39	45.19 ± 0.24	48.98 ± 0.11

6. Singlet oxygen quantum yield (Φ_Δ)

1,3-Diphenylisobenzofuran (DPBF) was used as a singlet oxygen scavenger for the singlet oxygen production by monitoring the absorbance of DPBF at 410 nm. The singlet oxygen quantum yield Φ_Δ was determined in an indirect method using methylene blue (MB; Φ_Δ = 52% in MeOH) as a standard and compared with rose bengal (RB). The Φ_Δ values were determined by the following equation.[11]

$$\Phi_\Delta^{sam} = \Phi_\Delta^{std}\left(\frac{F_{std}}{F_{sam}}\right)\left(\frac{k_{sam}}{k_{std}}\right)$$

$$F = 1 - 10^{-A}$$

Herein, sam and std represent the sample and standard, respectively. Φ_Δ is the singlet oxygen quantum yield, A is the absorbance at the excitation wavelength, k is the slope of the absorbance change of DPBF over time under photoexcitation.

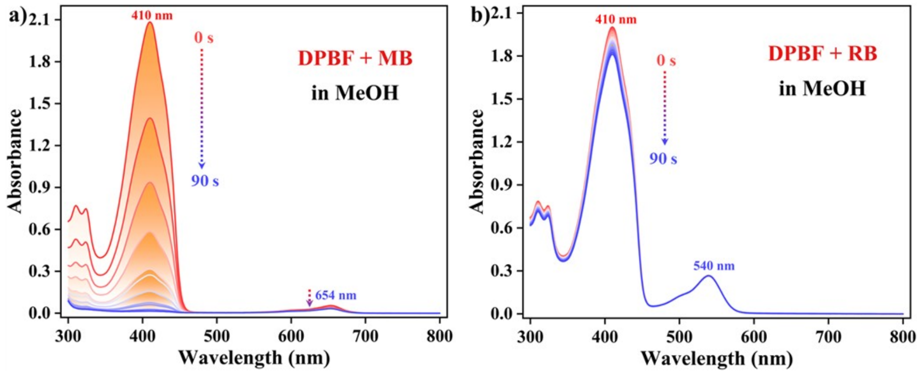

Fig. S14. Time-dependent UV-vis absorption spectral changes of MB/DPBF (a) and RB/DPBF (b) in MeOH during the irradiation time (t) of 90 s. *Notes*: 638 nm laser irradiation at a power density of 0.5 W/cm^2.

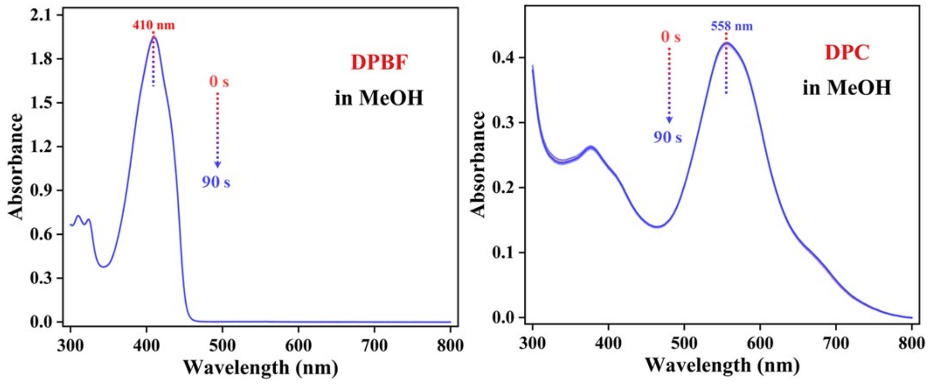

Fig. S15. Time-dependent UV-vis absorption spectral changes of DPBF (a) and DPC (b) in MeOH during the irradiation time (t) of 90 s. *Notes*: 638 nm laser irradiation at a power density of 0.5 W/cm^2.

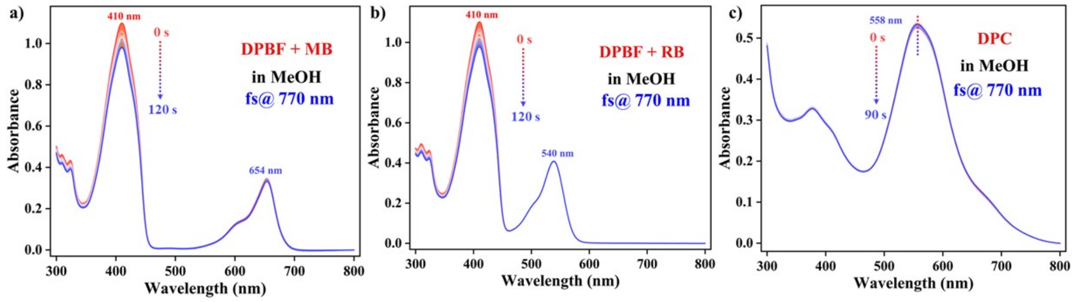

Fig. S16. Time-dependent UV-vis absorption spectral changes of MB/DPBF (a), RB/DPBF (b) and DPC (c) in MeOH during the irradiation time (t) of 120 s. *Notes*: 770 nm fs-laser irradiation at a power density of 0.2 W/cm^2.

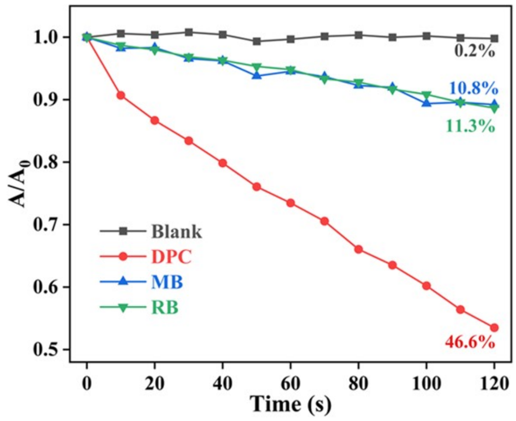

Fig. S17. Plots of A/A_0 versus laser irradiation times in the presence of different photosensitizers and DPBF. *Notes*: A_0 and A represented the absorbance of DPBF before and after laser irradiation.

Table S8. Calculation parameters for the singlet oxygen quantum yields.

Compounds	Φ_Δ	A_{410}	k	*F*
MB (standard)	52%	0.40	-0.087	0.088
DPC (in MeOH)	8.4%	0.109	-0.035	0.222

7. Femtosecond transient absorption (fs-TA) spectroscopy

The fs-TA setup used for this study was based on a PHAROS laser system with Light Conversion (1030 nm, <190 fs, 200 μJ/pulse, and 100 kHz repetition rate), nonlinear frequency mixing techniques and the Femto-TA100 spectrometer (Time-Tech Spectra). In brief, the 1030 nm output pulse of the regenerative amplifier was divided into two parts using an 80% beam splitter. The reflected part was used to pump an ORPHEUS Optical Parametric Amplifier which generates a wavelength-tunable laser pulse in the range of 300 nm to 15.0 μm. As outlined in Scheme S2,[12] the transmitted 1030 nm beam was split into two parts again. One part with less than 50% was

attenuated by a neutral density filter and focused into a YAG window, generating a white light continuum from 500 nm to 1600 nm used for probe beam. The probe beam was focused on the sample with an Ag parabolic reflector. After the sample, the probe beam was collimated and then focused into a fiber-coupled spectrometer with CMOS sensors and detected at a frequency of 10 kHz. The intensity of the pump pulse used in the experiment was controlled by a variable neutral-density filter wheel. The delay between the pump and probe pulses was controlled by a motorized delay stage. The pump pulses were chopped by a synchronized chopper at a frequency of 5 kHz and the absorbance change was calculated with two adjacent probe pulses. And the polarization of the pump pulse was set at the magic angle (54.7°) relative to the probe pulse. All experiments were carried out in 2.0 mm cuvettes at room temperature and the optical density of the samples was kept below 0.1 at the excitation wavelength.

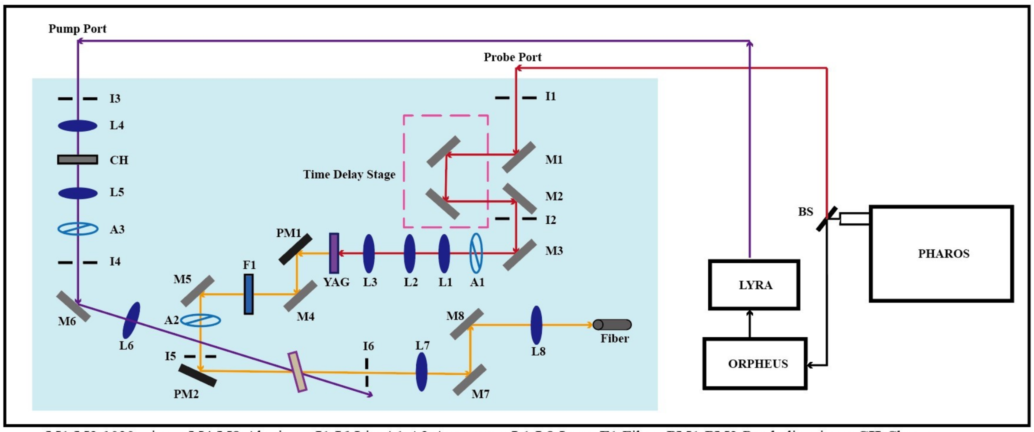

M1-M3:1030 mirror; **M4-M8:**Al mirror; **I1-I6:**Iris; **A1-A3:**Attenuator; **L1-L8:**Lens; **F1:**Filter; **PM1-PM2:**Parabolic mirror; **CH:**Chopper

Scheme S2. Schematic diagram of the setup of fs-TA spectroscopy.

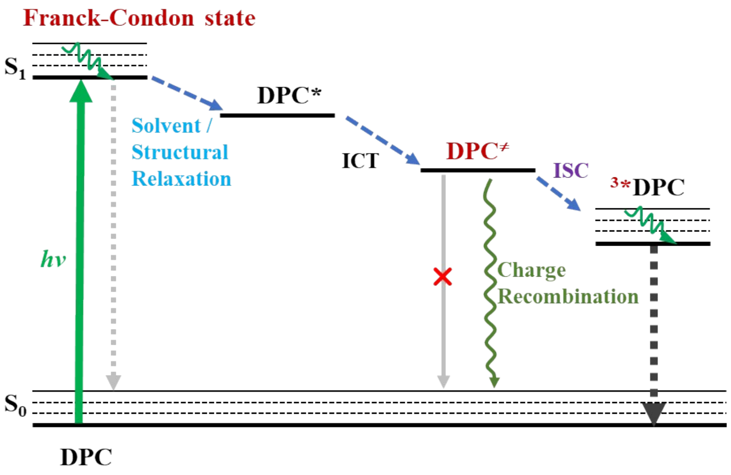

Fig. S18. Schematic diagram of the photoexcitation, intersystem crossing, and relaxation processes in the femtosecond time scale of DPC in MeOH excited at 540 nm.

8. Photothermal conversion studies

The photothermal conversion efficiency of DPC was generated by measuring the temperature change of DPC in

the water-DMSO solution (9/1, *v*/*v*) as a function of time under the continuous irradiation of 638 nm laser for 10 min (*t*) till the temperature of solution reached a steady-state case. The photothermal conversion efficiency (η) was calculated based on the following equation

$$\eta = \left[hA(T_{max} - T_{sur}) - Q_{dis}\right] / \left[I(1 - 10^{-A_\lambda})\right]$$

Here, h is the heat transfer coefficient, A is the surface area of the container, T_{max} is the maximum steady-state temperature, T_{sur} is the ambient temperature of the environment, Q_{dis} is the heat dissipation from the light absorbed by the solvent and the quartz sample cell, I is the incident laser power, and A_λ is the absorbance of the sample at 638 nm (0.405, 1.0 mm cuvette). The value of hA is calculated based on the following equation

$$hA = m_D c_D / \tau_s$$

Here, τ_s is the time constant for the heat transfer in the system, which was accessed based on the measurements in Figure 6d (τ_s = 302 s), and m_D and c_D are the mass (0.66 g) and heat capacity (4.2 J/g/°C) of the water, respectively. As a result, the hA was determined to be 9.17 mW/°C. Consequently, the calculated photothermal conversion efficiency of DPC in the water-DMSO solution was ~36.8%.

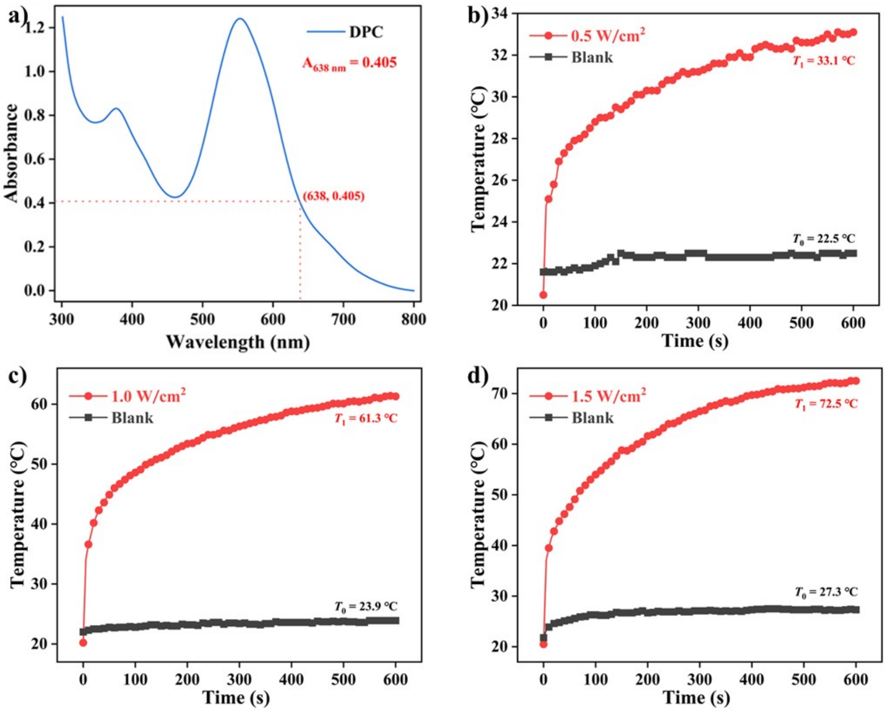

Fig. S19. a) UV-vis spectra of the phenanthroline-carbolong DPC in water-DMSO solution (9/1, *v*/*v*). *Notes*: c_{DPC} = 1.00 mg/mL; 1.0 mm length quartz cuvette. b) Photothermal heating curves of DPC (660 µL, 1.00 mg/mL) upon 638 nm laser irradiation with 0.5 W/cm^2 (b), 1.0 W/cm^2 (c), and 1.5 W/cm^2 (d) laser power densities.

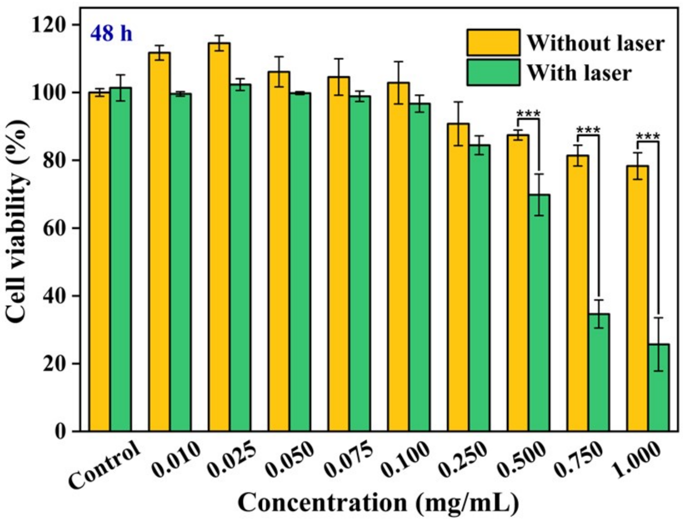

Fig. S20. Cell viabilities of 4T1 cells incubated with DPC at different concentrations (0.01 ~ 1.00 mg/mL) for 48 h without or with laser irradiation. *Notes*: 638 nm laser irradiation at a power density of 1.0 W/cm^2.

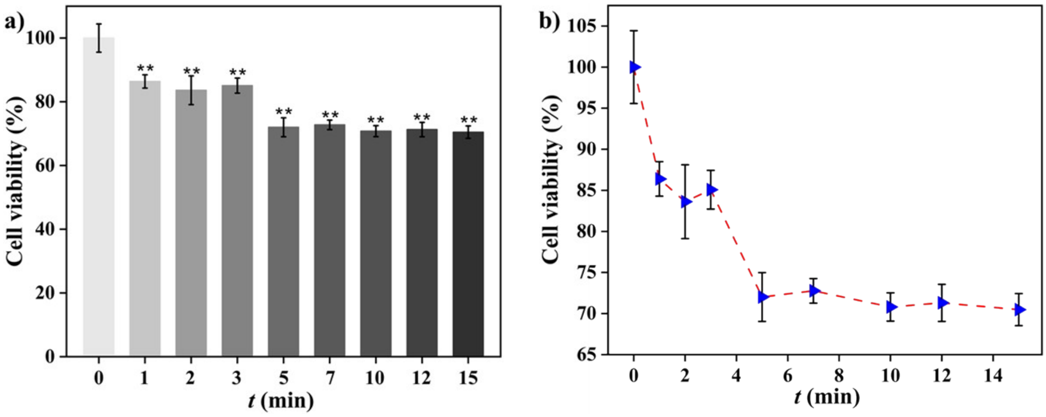

Fig. S21. Bar chart (a) and dot line (b) about cell viabilities of 4T1 cells incubated with DPC for different time (0 ~ 15 min, c_{DPC} ~ 0.5 mg/mL) with laser irradiation. *Notes*: 638 nm laser irradiation at a power density of 1.0 W/cm^2.

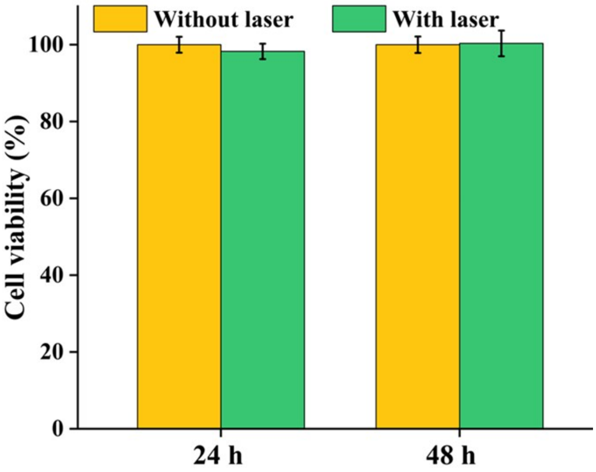

Fig. S22. Cell viabilities of 4T1 cells incubated without or with laser irradiation (5 min, 638 nm, 1.0 W/cm^2).

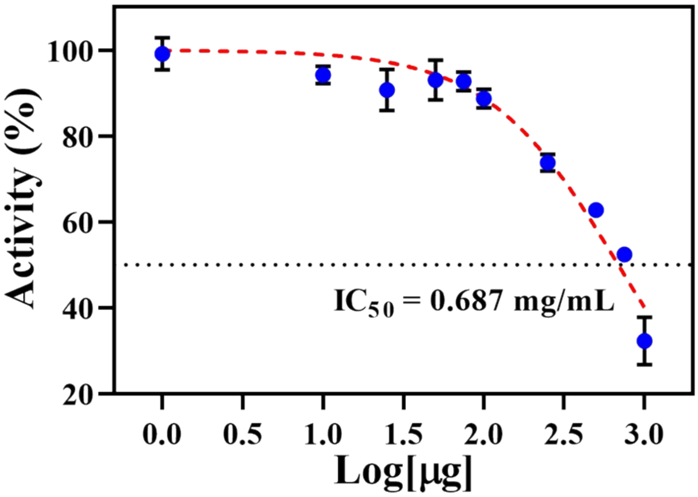

Fig. S23. IC_{50} of 4T1 cells incubated with DPC at different concentrations (0.01 ~ 1.00 mg/mL) for 24 h with laser irradiation. *Notes*: 638 nm laser irradiation at a power density of 1.0 W/cm^2.

References

[1] X. Lai, S. Chen, X. Gu, H. Lai, Y. Wang, Y. Zhu, H. Wang, J. Qu, A. K. K. Kyaw, H. Xia and F. He. *Nat. Commun.*, 2023, **14**, 3571.

[2] W. Feng, K. Liu, J. Zang, G. Wang, R. Miao, L. Ding, T. Liu, J. Kong and Y. Fang. *ACS Appl. Mater. Interfaces*, 2021, **13**, 28985−28995.

[3] J. Zang, W. Feng, X. Chang, K. Liu, H. Peng, L. Ding, T. Liu and Y. Fang. *Dyes Pigm.*, 2021, **193**, 109487−109493.

[4] M. Sheik-Bahae, A. A. Said, T. H. Wei, D. J. Hagan and Van E. W. Stryland. *IEEE J. Quantum Electron*., 1990, **26**, 760−769.

[5] M. Nikolay, D. Mikhail and R. Aleksander. *Opt. Express*, 2008, **16**, 4029−4047.

[6] A. Ajami, W. Husinsky, A. Ovsianikov and R. Liska. *Appl. Phys. B*, 2018, **124**, 142−151.

[7] C. Adamo and V. Barone. *J. Chem. Phys.*, 1999, **110**, 6158−6170.

[8] R. Ditchfield, W. J. Hehre and J. A. Pople. *J. Chem. Phys.*, 1971, **54**, 724−728.

[9] W. J. Hehre, R. Ditchfield and J. A. Pople. *J. Chem. Phys.*, 1972, **56**, 2257−2261.

[10] T. Lu and F. Chen. *J. Comput. Chem.*, 2012, **33**, 580−592.

[11] W. Yang, J. Zhao, C. Sonn, D. Escudero, A. Karatay, H. G. Yaglioglu, B. Küçüköz, M. Hayvali, C. Li and D. Jacquemin. *J. Phys. Chem. C*, 2016, **120**, 10162−10175.

[12] N. Zhang, L. Liu, H. Chang, K. Liu, T. Liu, L. Ding and Y. Fang. *J. Phys. Chem. Lett.*, 2023, **14**, 7283−7289.
